# Functional analysis of a novel mutation in the *TIMM8A* gene that causes deafness‐dystonia‐optic neuronopathy syndrome

**DOI:** 10.1002/mgg3.1121

**Published:** 2020-01-05

**Authors:** Addison Neighbors, Tonya Moss, Lynda Holloway, Seok‐Ho Yu, Fran Annese, Steve Skinner, Russell Saneto, Richard Steet

**Affiliations:** ^1^ Greenwood Genetic Center Greenwood SC USA; ^2^ University of South Carolina School of Medicine Columbia SC USA; ^3^ Program for Mitochondrial Medicine and Metabolism Division of Pediatric Neurology Neuroscience Institute Seattle's Children's Hospital University of Washington Seattle WA USA

**Keywords:** deafness‐dystonia‐optic neuronopathy syndrome Mohr–Tranebjaerg syndrome, mitochondrial inner membrane, *TIMM8A* gene, X chromosome

## Abstract

**Background:**

The rare, X‐linked neurodegenerative disorder, Mohr–Tranebjaerg syndrome (also called deafness‐dystonia‐optic neuronopathy [DDON] syndrome), is caused by mutations in the *TIMM8A* gene. DDON syndrome is characterized by dystonia, early‐onset deafness, and various other neurological manifestations. The *TIMM8A* gene product localizes to the intermembrane space in mitochondria where it functions in the import of nuclear‐encoded proteins into the mitochondrial inner membrane. Frameshifts or premature stops represent the majority of mutations in *TIMM8A* that cause DDON syndrome. However, missense mutations have also been reported that result in loss of the *TIMM8A* gene product.

**Methods:**

We report a novel *TIMM8A* variant in a patient with DDON syndrome that alters the initiation codon and employed functional analyses to determine the significance of the variant and its impact on mitochondrial morphology.

**Results:**

The novel base change in the *TIMM8A* gene (c.1A>T, p.Met1Leu) results in no detectable protein and a reduction in *TIMM8A* transcript abundance. We observed a commensurate decrease in the steady‐state level of the Tim13 protein (the binding partner of Tim8a) but no decrease in *TIMM13* transcripts. Patient fibroblasts exhibited elongation and/or increased fusion of mitochondria, consistent with prior reports.

**Conclusion:**

This case expands the spectrum of mutations that cause DDON syndrome and demonstrates effects on mitochondrial morphology that are consistent with prior reports.

## Graphical abstract

We report a novel TIMM8A variant in a patient with DDON syndrome that alters the initiation codon, resulting in no detectable protein and a reduction in TIMM8A transcript abundance. Decreased steady‐state level of the Tim13 protein and elongation and/or increased fusion of mitochondria were also observed in patient cells. This case expands the spectrum of mutations that cause DDON syndrome.

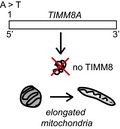

## 1 INTRODUCTION

Referred herein as DDON syndrome, the synonymous condition Mohr–Tranebjaerg syndrome (MTS) was first described nearly sixty years ago as an X‐linked condition affecting Scandinavian populations (Jin et al., 1996; Tranebjaerg, 1993; Tranebjaerg et al., 1995). Characterized primarily by progressive deafness in early childhood, this condition also manifests with dystonia, spasticity, and dysphagia (Bahmad, Merchant, Nadol, & Tranebjaerg, 2007; Ha et al., 2012; Kojovic et al., 2013). Mental disturbances and vision loss with variable onset and progression are also common phenotypes (Tranebjaerg et al., 2001). Located on Xq22, the gene associated with DDON syndrome, *TIMM8A* (originally called DDP for deafness–dystonia peptide; OMIM#300356) encodes a small protein that localizes to the intermembrane space in mitochondria (Jin et al., 1996; Tranebjaerg et al., 1995). Tim8 forms a complex with other small TIM proteins, to facilitate the import of nuclear‐encoded proteins into the mitochondrial inner membrane (Beverly, Sawaya, Schmid, & Koehler, 2008; Hasson et al., 2010; Rothbauer et al., 2001).

The pathogenetic mechanism of DDON is not fully defined. However, several studies have implicated impaired transport through the intermembrane space, and subsequent mitochondrial dysfunction when Tim8 is unable to associate with its binding partner, Tim13, as the primary driver of pathogenesis. Loss of the Tim8/Tim13 complex alters the transport and function of other proteins in the inner mitochondrial membrane, including Tim22 and Tim23 (Hasson et al., 2010; Rothbauer et al., 2001). Notably, downregulation or absence of Tim8 does not affect Tim23 import or levels in patient cell lines (Engl, Florian, Tranebjaerg, & Rapaport, 2012). This and other studies show that loss of *TIMM8A* results in abnormal mitochondrial morphology but this is not associated with any obvious impact on mitochondrial energetics (Binder et al., 2003; Engl et al., 2012). More recently, loss of Tim8a in neurons was shown to cause defects in Complex IV assembly, priming these cells for apoptotic vulnerability (Kang et al., 2019). Most of the mutations associated with DDON syndrome are frameshifts or premature stops, and there are a few missense mutations reported, including two in the first codon of the gene (Aguirre et al., 2006; Binder et al., 2003; Blesa et al., 2007; Hofmann et al., 2002; Penamora‐Destriza et al., 2015; Ujike, Tanabe, Takehisa, Hayabara, & Kuroda, 2001; Wang et al., 2019). Whether these first codon mutations result in the utilization of an alternate start site, or whether they result in the complete loss of protein noted in other DDON syndrome patients, is not known. In this report, we describe a male patient harboring a novel base change in the *TIMM8A* gene (c.1A>T, p.Met1Leu) with features of DDON syndrome and provide functional studies to confirm the pathogenic status of this variant.

### 1.1 Clinical summary

Our male patient was the product of a nonconsanguineous normal pregnancy. Term delivery was via C‐section due to placental hemorrhage and fortunately there were no perinatal problems from delivery. Prior to age 3 years, he met all developmental milestones on time without stagnation or regression. At 3 years, his parents began to notice some regression in his expressive language. He was eventually diagnosed with an auditory neuropathy and at age 6 years received cochlear implants. His most recent ophthalmological examination, at age 6, did not show optic neuropathy or retinal involvement. His neurological examination was otherwise without abnormality; in particular, findings of ataxia or dystonia were absent.

Due to early auditory neuropathy, a massively parallel gene sequencing panel was sent for commercial testing for hearing loss gene abnormalities (Prevention Genetics). A likely pathogenic variant in the *TIMM8A* gene, c.1A>T, p.Met1Leu was found. On parental testing, mother was found to be a carrier, with low copy numbers of the variant compared to wild type, suggesting that mother may be a mosaic for this genetic change.

## 2 METHODS

### 2.1 Patient information and ethical compliance

Informed consent was obtained from the parents of the proband prior to participation in the research study. All procedures employed were reviewed and approved by the appropriate institutional review committee in the GGC IRB protocol.

### 2.2 SDS‐PAGE and western blotting

Fractionated samples from both patient and control cell lines were resolved on a 15% SDS‐PAGE gel followed by transfer to nitrocellulose membranes and blocked using 5% nonfat powdered milk in TBS‐T solution for 1 hr at room temperature. The following primary antibodies were utilized at specific dilutions as indicated: Proteintech rabbit polyclonal TIMM8A antibody at (1:1,000, 24 hr, 4°C); Santa Cruz mouse monoclonal GAPDH (6C5) antibody (1:10,000, 30 min, room temperature); Santa Cruz mouse monoclonal Lamin A/C (636) antibody (1:500, overnight, 4°C); Santa Cruz mouse monoclonal Cytochrome C (7H8) antibody (1:200, overnight, 4°C); and Proteintech rabbit polyclonal TIMM13 antibody (1:500 dilution, 12 hr, 4°C). Secondary antibodies used were anti‐mouse‐HRP (1:3,000, 1 hr, room temperature) and anti‐rabbit‐HRP (1:1,000, 1 hr, room temperature). Ponceau S staining was performed to visualize total protein load.

### 2.3 Isolation of nuclear/mitochondria by subcellular fractionation

A subcellular fractionation protocol was utilized in order to enhance the signal of the TIMM proteins trapped within the mitochondrial intermembrane. Patient cells lines were cultured, fractionated into two lysates, with the first containing both nuclei and mitochondria and a second lysate including cytoplasmic and cellular membrane contents. All steps were performed on ice and centrifugation at 4°C. Subcellular fractionation buffer (SF buffer) containing 250 mM sucrose, 10 mM KCl, 20 mM HEPES (pH 7.4), 1.5 mM MgCl_2_, and 1 mM EDTA, 1 mM DTT, and protease inhibitor cocktail, was added to fibroblasts and lymphoblasts to generate the cell homogenates. Homogenates were passed through a 25‐gauge needle ten times before centrifugation at 8,000 rpm (10,000 *g*) for 12 min resulted in a pellet containing the nuclear and mitochondrial material and a supernatant fraction. RIPA buffer was added to the fractionated cellular components to solubilize the proteins, and a BCA assay performed to quantify total protein concentration in each fraction.

### 2.4 Mitochondrial staining

Fibroblasts were seeded onto coverslips in a twelve‐well cell culture plate. After washing with DPBS containing calcium and magnesium, the cells were fixed to the slides with the addition of 3.7% formaldehyde for 10 min followed by washing 4 times in DPBS containing calcium and magnesium. Permeabilization was achieved with the addition of 1 ml of 0.1% Triton‐X in DPBS for 10 min at room temperature, and then, wells were washed three times with DPBS containing calcium and magnesium. Fixed coverslips were blocked using 3% bovine serum albumin and incubation for 1 hr at room temperature on a rotator followed by washing with DPBS. A rabbit polyclonal anti‐P5CS (ALDH18A1) antibody (1:250 dilution) was used to label mitochondria in staining buffer for one hour at room temperature followed by incubation with an Alexa Fluor 488 goat anti‐rabbit IgG secondary antibody (Abcam, 1:500 dilution) in the dark for 1 hr. After washing, the coverslips were mounted with Prolong Gold^TM^ and visualized using an Olympus FV3000 confocal microscope. To quantify the percentage of elongated mitochondria, images from 8 different fields of cells were obtained and the percentage of elongated mitochondria relative to the total mitochondria scored was determined in each field. These percentages were then averaged, and statistical significance calculated using a Student's *t* test.

## 3 RESULTS

### 3.1 Genetic and biochemical characterization

**Figure 1:**
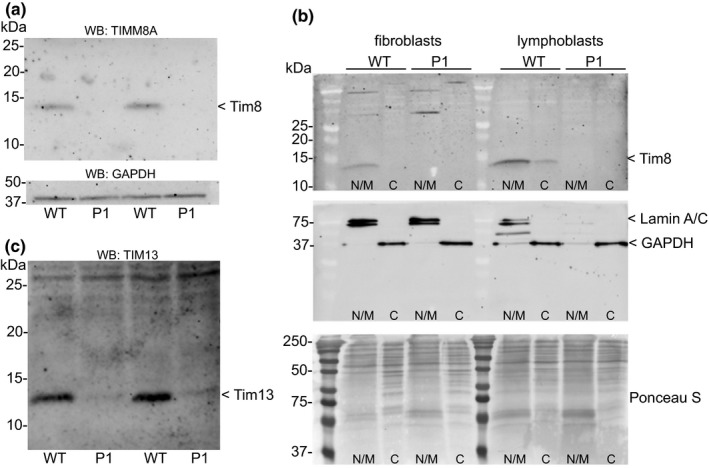
(a) Western blot analysis of the *TIMM8A* gene product in control (WT) and patient (P1) fibroblast lysates. A representative blot of three independent experiments is shown. GAPDH was used as a loading control. (b) Subcellular fractionation and Western blot analysis of fibroblast and lymphoblast lysates. Lamin A/C and GAPDH were used as controls for the fidelity of the fractionation of nuclear/mitochondria and cytosolic pools, respectively. (c) Western blot analysis of the *TIM13* gene product in control (WT) and patient (P1) fibroblast lysates. Tim13 protein is greatly reduced but still detectable in patient cells

**Figure 2:**
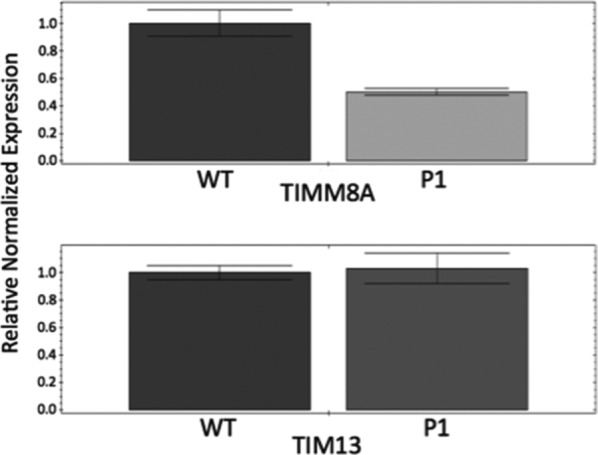
Quantitative PCR analysis of *TIMM8A* and *TIM13* transcript abundance in WT and P1 fibroblasts, normalized to *RPL4* transcript abundance. Data represent the average of three independent analyses

Patients with DDON syndrome typically bear loss of function mutations in the *TIMM8A* gene, although some missense mutations have also been reported that impact the start codon. Massive parallel sequencing using an auditory neuropathy panel and buccal cells of the patient uncovered an A>T change in the initiation codon, which can in theory alter both translation initiation and possibly *TIMM8A* transcription. To study whether the patient's *TIMM8A* mutation impairs translation of the protein, we first performed Western blot analysis on patient fibroblasts. The results of this experiment show that the patient's fibroblasts make no detectable Tim8a protein, consistent with the fact that the mutation disrupts the start codon of the *TIMM8A* transcript (Figure 1a). There was no evidence of a truncated peptide in these cells that might indicate the usage of an alternate start site, although we cannot rule out that another start site is used but produces an unstable peptide. We also examined Tim8a levels in patient lymphoblasts to determine whether loss of the protein was observed in another cell type. Both fibroblasts and lymphoblasts were lysed using a detergent‐free, subcellular fractionation buffer, and the mitochondria and nuclei fraction separated from the cytosolic fraction. Analysis of marker proteins for the nuclear/mitochondrial fraction (Lamin A/C) and cytosol (GAPDH) demonstrate the fidelity of the fractionation, and Ponceau S staining shows an equivalent amount of total protein was resolved. The results in Figure 1b show that regardless of the cell type, the patient does not make any detectable levels of the Tim8 protein. We next assessed whether loss of the Tim8a protein would alter the steady‐state level of its binding partner Tim13. Prior reports demonstrated variable loss of Tim13 in DDONS/MTS patient cells. Western blot of fibroblast lysates showed a striking reduction, but not absence, of Tim13 in the patient cells (Figure 1c). The reduction of Tim13 is at the level of the protein as qPCR analysis revealed equivalent transcript abundance in control and patient fibroblasts (Figure 2). However, *TIMM8A* transcript was reduced roughly 50% in the patient fibroblasts, suggesting that the A>T base change may impact the transcription of the *TIMM8A* gene.

**Figure 3:**
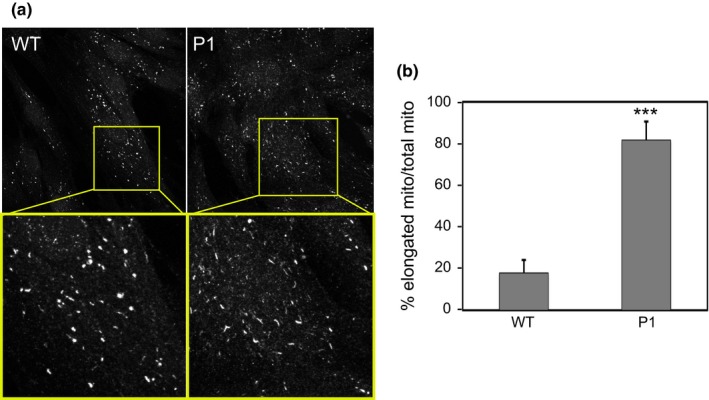
(a) Immunostaining of the mitochondrial enzyme pyrolline 5‐carboxlate synthase (P5CS) in WT and P1 fibroblasts. (b) Quantification of the percentage of elongated mitochondria relative to the total mitochondria in eight cell fields across two independent experiments. 60–80 mitochondria per cell field were counted. ***p < .001

Tim8a and Tim13 are part of a small protein complex involved in the import of proteins through the inner mitochondrial membrane. Loss of this import has been shown to cause altered mitochondrial morphology (Binder et al., 2003; Engl et al., 2012). Using an antibody to the mitochondrial enzyme, pyrroline 5‐carboxlate synthase (P5CS), we stained control and patient fibroblasts to ask whether morphology of this organelle was abnormal (Figure 3). Our results show a clear increase in elongated mitochondria in the patient. These elongated mitochondria, which have a cigar‐like (as opposed to a globular) appearance in the patient cells, may arise due to increased fusion—a phenotype that could reflect the failure to import specific inner membrane proteins or another Tim8‐dependent function.

## 4 DISCUSSION

With the addition of a novel frameshift variant described in 2019 (Wang et al., 2019), approximately fifteen pathogenic variants in the *TIMM8A* gene resulting in DDON have been documented in the literature. The variant described in this report of a c.1A>T start loss bears similarity to earlier start loss cases (Binder et al., 2003; Merchant et al., 2001; Penamora‐Destriza et al., 2015; Tranebjaerg, 2012). In all three cases, the methionine required to initiate protein translation is replaced leading to a complete absence of the Tim8a/DDP protein. The absence or variant forms of the *TIMM8A* gene product appear to only significantly influence the function of neurons within specific neuronal populations. Roesch et al. revealed elevated levels of both Tim8 and Tim13 within large neurons of the brain in mouse models of specific structures including components of the basal ganglia, brainstem, and the Purkinjie cells of the cerebellum (Roesch, Hynds, Varga, Tranebjaerg, & Koehler, 2004). Implication of these structures may correlate with the symptomatology in DDON patients, including the characteristic dystonia and variable neuropsychological features.

Rates of mitochondrial fusion and fission directly dictate the organelle's morphology and function. The balanced intramitochondrial contents achieved by proper fission and fusion are required for normal functioning (Chen et al., 2010). Beyond its function of shuttling proteins across the mitochondrial intermembrane space, Arnoult et al. characterized the role of the C‐terminal portion of Tim8a interacting with the well‐characterized dynamin‐related protein 1 (DRP1) during programmed cell death (PCD) of the mitochondria (Arnoult et al., 2005). They demonstrated extensive fragmentation of mitochondria occurs during PCD, which is accomplished through increased rates of fission when DRP1 localizes to the mitochondria via its interaction with Tim8a. These authors concluded that this DRP1‐mediated mitochondrial fission occurs during caspase‐independent apoptosis. HeLa cells harboring a dominant‐negative mutant of DRP1 demonstrated an elongated morphological appearance with fused interconnected tubules because of the inhibition of mitochondrial fission (Arnoult et al., 2005). Engl et al. published work documenting that both DDON patient fibroblasts featuring the c.116delT variant in the *TIMM8A* gene and HeLa cells transfected with siRNA targeting *TIMM8A* yielded mitochondrial with similar elongated morphology (Engl et al., 2012).

Neurons function as highly polarized cells that require a sufficient amount of mitochondria within specific locations to carry out its actions such as axonal transport and synaptic transport. This polarity requires DRP1‐directed mitochondrial fission during development to appropriately distribute mitochondria and represents an essential requirement for neuronal viability. While expendable in most other cells of the body such as fibroblasts, DRP1 depletion prevents mitochondria survival and proliferation in the developing neuron so that their processes fail to generate the appropriate synaptic formation with other neurons (Kageyama et al., 2012). Additionally, it was shown that mitochondrial fission enables postmitotic neuron survival for a short time by reducing damage by oxidative species. Purkinjie cells of the cerebellum in DRP1 deficient mice increased fusion and caused mitochondria tubules to elongate. Likewise, hippocampal primary neurons in rats with suppressed DRP1 expression slowed cell death and fragmentation of mitochondria when compared to overexpression of DRP1, but ultimately did not protect against necrotic death of the neuron (Estaquier & Arnoult, 2007).

Based upon these findings, one theory to account for DDON syndrome pathogenesis would be that the primary neurons of these sensory pathways along with other cortical tracts require an advanced level of regulation of their mitochondrial morphology via the Tim8 and DRP1 interaction to ensure the appropriate distribution of mitochondria as these primary neurons further differentiate. Failure of proper localization of DRP1 to the mitochondria due to the absence of the Tim8 protein leading to subsequent loss of mitochondrial fission would cause an accumulation of elongated mitochondria. Further differentiation of these primary neurons as the brain matures would not allow for proper recycling of mitochondria so that accumulation of damaged mitochondria would lead to death of these primary neurons, mirroring the postmortem findings described above. This Tim8/DRP1 interaction must play a central role in the primary neurons of the auditory and visual sensory pathways so that in its absence, degeneration occurs to produce the hallmark DDON symptomology and histopathologic findings.

Optic atrophy protein 1 (Opa1) is a well‐characterized mediator of mitochondrial fusion that is localized to the mitochondrial inner membrane. Its function is in direct opposition to that of DRP1 and contains splice variants that are specific to tissues located in the retina and cochlea. Mutations in Opa1 resulting in autosomal‐dominant optic atrophy plus syndrome (ADOA+) bear a striking resemblance to that of DDON (Davies et al., 2007; Yu‐Wai‐Man, Griffiths, & Chinnery, 2011; Yu‐Wai‐Man et al., 2010). Here, however, optic atrophy with visual impairment is the initial manifestation during childhood, followed by sensorineural hearing loss in late childhood to young adulthood. Mitochondria in ADOA+ display morphological abnormalities characterized by increased fragmentation resulting in smaller, more punctuated mitochondria. This morphological difference is attributed to increased rates of mitochondrial fission due to the lack of Opa1 mediated fusion. This has been demonstrated in both retinal and nonretinal cells such as fibroblasts (Davies et al., 2007).

Advancements in the therapies that hold the potential to treat single gene‐causing disorders represent an exciting frontier in clinical research. DDON syndrome results from mutations of a single gene, *TIMM8A*, and the novel variant described in this case report as a start loss located at the first codon may be a candidate for read through pharmaceuticals (Bello & Pegoraro, 2016; Keeling, Xue, Gunn, & Bedwell, 2014; Rowe & Clancy, 2009). These drugs are characterized by binding to ribosomes to induce a translational “readthrough” of premature stop codons to generate a full‐length protein. The drug ELX‐02 is an investigational synthetic eukaryotic ribosome–selective glycoside currently undergoing clinical trials to assess its use in CF patients currently within the United States (Leubitz et al., 2019). Whether this compound, or other drugs being considered for the treatment of optic neuropathy, will prove effective requires further investigation.

## CONFLICT OF INTEREST

The authors declare no conflict of interest as part of this work. This manuscript does not contain any shared data.
